# Systematic literature review of IL-6 as a biomarker or treatment target in patients with gastric, bile duct, pancreatic and colorectal cancer

**DOI:** 10.18632/oncotarget.25661

**Published:** 2018-07-03

**Authors:** Noomi Vainer, Christian Dehlendorff, Julia S. Johansen

**Affiliations:** ^1^ Department of Oncology, Herlev and Gentofte Hospital, Copenhagen University Hospital, Copenhagen, Denmark; ^2^ Statistics and Pharmacoepidemiology, Danish Cancer Society Research Center, Copenhagen, Denmark; ^3^ Department of Medicine, Herlev and Gentofte Hospital, Copenhagen University Hospital, Copenhagen, Denmark; ^4^ Institute of Clinical Medicine, Faculty of Health and Medical Sciences, University of Copenhagen, Copenhagen, Denmark

**Keywords:** biomarker, gastrointestinal cancer, interleukin-6, therapeutic target

## Abstract

Gastrointestinal cancer (GI) is a major health problem. Patients with gastric, pancreatic, colorectal, bile duct and gall bladder cancer often have advanced disease at the time of diagnosis and are generally difficult to cure, resulting in a dismal prognosis for most patients. Inflammation plays an important role in the development and growth of cancer, which has led to a growing interest in the pro-inflammatory cytokine interleukin 6 (IL-6).

The aim of the present review was to evaluate the clinical use of IL-6 as a biomarker or therapeutic target in patients with GI cancer. We did a systematic review of studies (1993–2018), to assess the clinical use of IL-6 as a diagnostic, prognostic or predictive tumor biomarker or as a potential therapeutic target.

This review includes 48 studies and 5316 patients. Circulating IL-6 levels appear to be an independent prognostic biomarker in patients with GI cancer, with high IL-6 levels associated with short overall survival (OS). The results for colorectal cancer were too ambiguous to give conclusive results. IL-6 seemed to be a marker for some of the clinical characteristics of GI cancer, and may have a role in the diagnostic workup in general practice. No published studies have examined the use of IL-6 as a therapeutic target in pancreatic, gastric, bile duct or colorectal cancer.

In conclusion, high circulating IL-6 was associated with short OS in most studies in GI cancer patients. Whether inhibition of IL-6 would decrease GI cancer symptoms and increase quality of life is unknown.

## INTRODUCTION

Gastrointestinal (GI) cancer accounted for approximately 2.85 million new cases and 1.89 million deaths world-wide in 2012 [1]. In this study, we focus on gastric cancer, bile duct cancers, pancreatic cancer and colorectal cancer. These patients often present with locally advanced or metastatic disease at the time of diagnosis, and are therefore not candidates for curative surgery [2–7]. The prognosis is often dismal, particularly for patients with gastric cancer, pancreatic cancer and bile duct cancer, which includes cholangiocarcinoma and gallbladder cancer. The 5-year overall survival (OS) is only 10–20% for gastric cancer [7, 8], 15% for bile duct cancer [9], 3–7% for pancreatic cancer [5, 6, 10–12], and 50–65% for colorectal cancer [3, 13]. In 2015, colorectal cancer was the second most common cancer in women, third in men, while it is the fourth leading cause of cancer deaths in men and the third in women [3, 14, 15]. Worldwide, gastric cancer is the fourth most common cancer and the third most common cause of cancer death [1, 15]. Today, pancreatic cancer is the seventh most common cause of death from cancer [1], and in 2030, pancreatic cancer is predicted to become the second leading cause of death from cancer [16]. GI cancers are also among the most common types of cancer, since colorectal and pancreatic cancer both were among the most common cancers in 2016 together with breast cancer, lung and bronchial cancer and prostate cancer [17].

Screening programs for early detection of some GI cancers are available in some countries. In Japan and South Korea, countries with a very high incidence of gastric cancer, national screening programs using endoscopy and photofluorography are used to detect gastric cancer [8]. However, these screening programs are expensive and are only cost-effective in moderate- to high-risk populations. A number of non-invasive screening techniques are currently being assessed, including gastric-specific pepsinogen and gastrin-17 [8]. Screening for colorectal cancer by feces sampling and colonoscopy is used in many countries [18, 19], presumably resulting in colorectal cancer being diagnosed 2 to 3 years earlier than without screening. However, the screening may result in overtreatment, given that most adenomas never develop into cancer [2].

New biomarkers for the early detection and evaluation of prognosis for patients with GI cancers are therefore pivotal [20, 21]. A tumor biomarker indicates that a tissue has become malignant or indicates the likely behavior of the tumor in terms of prognosis or response to therapy [22]. Very few biomarkers are currently used in daily clinical practice. Serum levels of carcinoembryonic antigen (CEA) are used to follow treatment efficacy in patients with gastric and colorectal cancer and to monitor gastric and colorectal cancer patients after surgery to detect recurrences early [23]. Serum CEA is also used as a stage-dependent prognostic biomarker in patients with colorectal cancer, but its clinical usage is hampered by a low sensitivity [24]. Serum levels of cancer antigen 19.9 (CA 19.9) are used to follow treatment efficacy in patients with pancreatic cancer and colorectal cancer, and after surgery to identify recurrences early, but serum CA 19.9 is neither sufficiently sensitive nor specific [4, 25]. Serum CA 19.9 is also a prognostic biomarker and associated with disease burden in pancreatic cancer patients, but not all patients with pancreatic cancer have elevated levels of serum CA 19.9 [5, 26, 27].

Recently the cytokine interleukin-6 (IL-6) has been subject to increased attention due to its possible usefulness as a biomarker of inflammation. This review aims to investigate whether circulating levels of IL-6 can be used as a diagnostic or prognostic biomarker in patients with GI cancer and to assess its potential as a therapeutic target.

### Interleukin-6

IL-6 is a pleiotropic, pro-inflammatory cytokine involved in many biological processes, including cancer and autoimmune diseases. The protein is a 21–28 kDa 4-helix bundled glycoprotein consisting of 184 amino acids [28–30]. IL-6 is produced by various normal cell types such as macrophages, monocytes, stromal cells, hematopoietic cells, epithelial cells and muscle cells, playing an important role in inflammation, immunity, reproduction, metabolism, hematopoiesis, neural development, bone remodeling and angiogenesis [31, 32]. In the tumor microenvironment, IL-6 is produced by multiple cell types including tumor infiltrating immune cells, fibroblast stromal cells and the tumor cells themselves, induced by various factors such as NF-κB, prostaglandin E2 (PGE_2_), interleukin-β (IL-1β), hypoxia, the lack of STAT3-inhibitors such as SOCS, microRNAs (miRNA) among others [29–31, 33–35]. IL-6 is part of a complex mixture of cytokines and chemokines in the tumor microenvironment [36]. IL-6 executes its function by binding to its receptor (R), either through the membrane bound receptor (mIL-6R) called the classical pathway, or through the trans-signaling pathway by binding to the soluble receptor (sIL-6R) in the tissue [30, 37]. In the classical pathway, IL-6 binds the mIL-6R, which is largely restricted to a small number of cells (leukocytes, megakaryocytes, hepatocytes and certain epithelial cells), and is important for the acute-phase response, hematopoiesis and homeostatic processes. When binding to the mIL-6R, the IL-6-mIL-6R-complex dimerizes, binds and activates the trans-cellular gp130. In the trans-signaling pathway, IL-6 binds to sIL-6R, the IL-6-sIL-6R-complex dimerizes and binds to the trans-cellular gp130, hence activating it. The trans-signaling pathway is particularly important in inducing the tumor-microenvironment, controlling leukocyte recruiting and the activation of tumor-associated stromal cells. Since gp130 is not restricted to specific cell types, the trans-signaling pathway can occur in the majority of cells [36]. In both pathways, gp130 is activated, which leads to the activation of gp130-associated JAKs (JAK1, JAK2, TYK2) to bind to the Box-domains in the gp130 protein, leading to a transphosphorylation and full activation of JAKs. Downstream, the STAT-family proteins are now capable of recognizing and binding to the docking sites of the activated JAKs, in order for JAK to phosphorylate and activate STAT [38]. In terms of malignancies, STAT3 is the more important member of the STAT-family inducing tumor growth and immunosuppression [29]. The activated STAT3 results in dimerization of the STAT3, which translocates to the nucleus and binds promoter regions of target genes, leading to secretion of pro-inflammatory factors and acute-phase proteins (e.g. IL-1β, IL-8) and resulting in an anti-apoptotic state of the cell inducing cell survival (cyclin D1, MYC, Bcl-x_L_ and survivin) [29, 31, 39], angiogenesis (VEGF) and invasion (matrix metalloproteases) [29], and secreting immunosuppressive factors (IL-10, TGFβ and VEGF) [28, 29] increasing chances for completing the cell cycle and cell and tumor growth [30, 31, 40–43]. Hence, high IL-6 levels promote an anti-apoptotic, proliferative state in tumor cells, thereby enabling the tumor cells to become anti-cancer drug resistant to both chemotherapy and cancer immunotherapy [38, 40, 41]. IL-6 has been shown to play an important role in regulating the proliferation and differentiation of T and B lymphocytes and natural killer cells [44]. In a recent study, MHC class II expression of Th1 cells was blocked by IL-6, hampering the secretion of IFN-γ and IL-2, thereby reducing the cytotoxic T-lymphocyte activity, enabling the cancer cells to evade the anticancer immunological reaction [37].

Various factors increase such as growth factors in the microenvironment (IL-1β, NF-κB, PGE_2_, low O_2_) and/or their active receptors, and the lack of STAT3 inhibitors increase the secretion of IL-6 [29]. Recently, a paracrine function of miRNA has been discovered, contributing to the idea of a positive feedback loop effect in the IL-6/JAK/STAT-cascade. Dendrite cells derived from colorectal tissue has been found to secrete miRNA-21 and -29b, inducing the production of IL-6. IL-6 itself induces more miRNA-release. MiRNA binds the TLR8 receptor of the immune cells, inducing more IL-6 and other cytokines as well as increasing invasion of the tumor cells *in vitro* [36].

IL-6 is a major mediator of inflammation, and it is important to elucidate that IL-6 is part of a complex, interdepending network of cytokines released in inflammatory conditions [36]. IL-6 is particularly important in chronic inflammatory conditions such as rheumatoid arthritis, inflammatory bowel disease, Castlemann’s disease, haematopoietic diseases and after physical stress such as surgery or chemotherapy. The anti-IL-6R antibody Tocilizumab is already approved in the treatment of patients with rheumatoid arthritis [45]. Increased levels of serum and/or tumor IL-6 are also seen in a number of malignant conditions, both haematopoietic malignancies and solid tumors including breast, cervical, esophageal, head-and-neck, ovarian, prostate, colorectal, pancreatic, hepatocellular, gall bladder, non-small-cell lung cancer and multiple myeloma, reflecting the immunological involvement in cancer. Several studies have shown IL-6 to be a prognostic indicator of survival as well as predictive in response to therapy in many types of cancer [29, 46]. A high IL-6 level is generally associated with a poorer outcome, particularly regarding renal cell, ovarian and prostate cancer, and correlated to more severe symptoms in regards to cancer as well as the development of anti-cancer drug resistance [12, 30, 39, 42, 44, 47–50].

### Inflammation and the development of cancer

Inflammation is often associated with the development of cancer, and GI cancers seem to be particularly sensitive to inflammation [51, 52]. Inflammation can both initiate and accelerate the development of the cancer lesions, giving rise to malignant formation. In GI cancers, the activation level of STAT3 seems to be of importance with regard to increasing tumor size and proliferation in mouse models, and IL-6 seems to be the most important activator of the STAT3-cascade [52, 53]. It is well documented that inflammation is related to the development of gastric cancer, an important risk factor being the bacterium *Helicobacter pylori,* even though the precise biological mechanism is unknown [21, 54, 55]. It is believed that *Helicobacter pylori* changes the expression of miRNAs and IL-6, which in turn downregulate other important tumor suppressor proteins such as p53 and the protein CDX2. In a study by Chung *et al.* [56], the treatment of gastric cancer cells with *Helicobacter pylori* was shown to regulate miRNA, particularly enhancing the expression of miR-195 and miR-488, which plays an important role in controlling IL-6. Saito *et al.* [57] showed that CDX2 is suppressed by the activation of the IL-6/STAT3 signal pathway via miR181b *in vitro*. CDX2 is involved in the intestinal cell differentiation in normal cells, and low CDX2 in tumor tissue is associated with a poorer cancer-specific survival. Hence, there is an increasing interest of the role of miRNA in the development of GI cancer. According to the EPIC-Eurogast study, 93% of patients with non-cardia gastric cancers were infected with *Helicobacter pylori* [58]. This bacterium causes a chronic inflammatory state which results in increased reactive oxidative stress, atrophy, intestinal metaplasia, dysplasia of gastric mucosa cells and increased methylation silencing tumor suppressor genes [7, 8, 59]. Reflux (Barrett’s esophagus) also predisposes to gastric cancer development [7].

A close relationship between chronic inflammation in the bile duct and cholangiocarcinoma has also been suggested. This is seen in primary sclerosing cholangitis, in which the risk of developing cholangiocarcinoma is 8–12% [4, 60–62]. In pancreatic cancer, inflammation also seems to be concurrent with the precursor lesions of pancreatic cancer, i.e. pancreatic intraepithelial neoplasia [11, 12, 63]. Chronic pancreatitis is an established risk-factor for pancreatic cancer, and patients suffering from inflammatory bowel disease have a 10-fold increased risk of developing pancreatic cancer [11, 12]. Inflammatory bowel disease is also an important risk factor in colorectal cancer with more than 20% developing colorectal cancer within 30 years of disease onset if the disease is poorly controlled [14, 64, 65].

Many studies have investigated the association between IL-6 and GI cancers, as well as other cancer types, resulting in an acknowledgment of a certain effect of IL-6 in developing and sustaining the neoplastic cells. Gastric cancer cells secrete IL-6, and an increase of IL-6 in serum and gastric cancer tissue appears to regulate tumor growth and development in an autocrine loop [59, 66–70]. Several *in vitro* cell studies have been performed regarding cholangiocarcinoma [43, 60, 62, 71], and all indicate that IL-6 has a pivotal effect on the growth and chemo-resistance of cholangiocarcinoma cells. IL-6 is secreted by cholangiocytes, which suggests that IL-6 is both a paracrine and an autocrine growth factor for normal cholangiocytes [60, 62, 72]. The increase of IL-6 in both inflamed and neoplastic cholangiocytes and the elevated serum IL-6 in patients with cholangiocarcinoma indicate that IL-6 is a key mediator of bile duct epithelial disorders, by inducing proliferation (MAPK pathway) and upregulating epithelial-to-mesenchymal transition (STAT3) and anti-apoptotic Bcl-2 molecules in cholangiocytes (Akt-dependent pathway) [43, 60, 71]. Anti-IL-6 was shown to increase the sensitivity to chemo-therapy [60], and decrease proliferation [62].

Regarding pancreatic cancer, IL-6 protein expression in pancreatic cancer cells is significantly increased compared to normal pancreatic cells [34, 73, 74]. Recently, a murine study has been performed investigating the effect of IL-6 receptor blockade on spontaneously arising tumors [49]. In combination with chemotherapy, IL-6 receptor blockade induced tumor cell apoptosis, tumor regression and improved OS. In a study using an orthotopic xenograft model with pancreatic cancer cells [63], treatment with Tocilizumab (anti-IL-6R) resulted in a remarkable decrease in tumor weight and new metastases compared to a control group. In a murine study that combined anti-IL-6 and anti-programmed death-1-ligand in treating pancreatic cancer [75], a decrease in tumor weight was observed compared to the control group and mice treated with either drug alone, and the OS improved by 35% compared to the control group. The authors also highlighted the tolerability of the anti-IL-6 drug, as they observed no change in body weight.

Non-colitis-associated colorectal cancer displays large numbers of inflammation cells within the solid tumor, which causes the production of mutagens (e.g. ROS and NO), tissue injury and pro-inflammatory cytokines, including IL-6 [64, 76]. IL-6’s activation of STAT3 appears to protect the epithelium of the GI tract from apoptosis and stimulates regeneration [53, 64, 77], and high IL-6 levels have been associated with increased colorectal cancer tumor risk [65]. Ying *et al.* [78] showed that IL-6 plays a pivotal role in the development of cancer stem cells in colorectal cancer and that anti-IL-6-antibody significantly increased chemo sensibility, and Cross-Knorr *et al.* [13] showed that apoptosis of murine colorectal cancer cells induced by oxaliplatin was lowered from 32% to 19% when colorectal cancer cells were co-treated with IL-6.

## REVIEW CRITERIA

**Figure 1:**
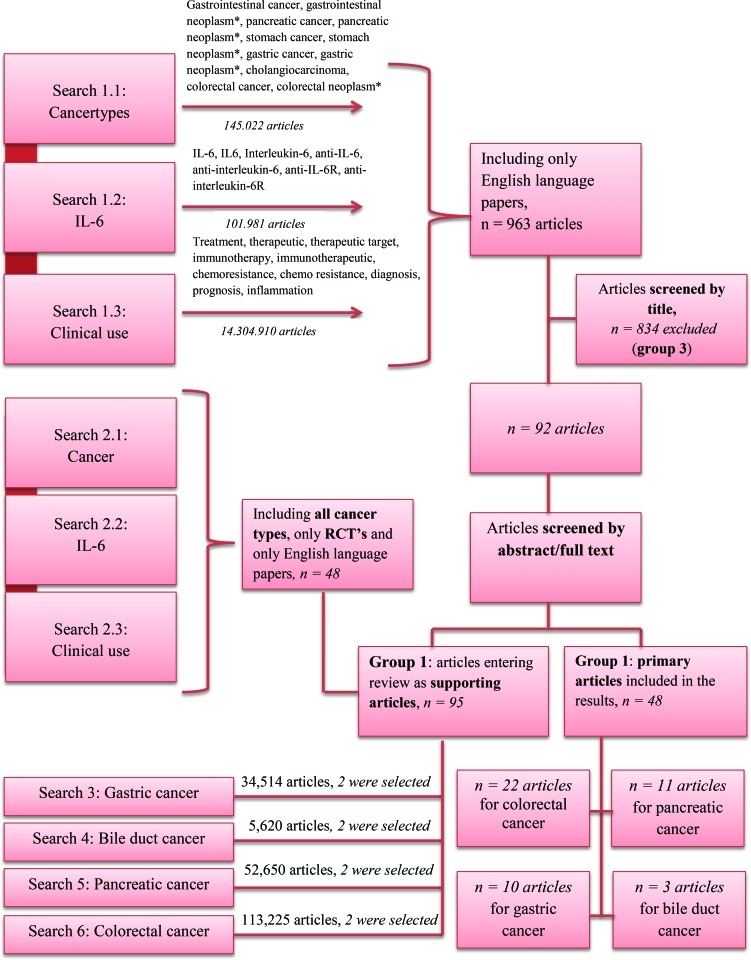
Pubmed search The review was based on six different searches using the search criteria shown in the Figure. The search criteria were combined as shown searching PubMed. Search 1 resulted in 963 articles, which were sorted by assessing the relevance of the abstract followed by a systematic reading of all non-excluded articles. Search 2 resulted in 48 articles, also sorted by assessing the relevance of the abstract followed by a systematic reading of all non-excluded articles. Search 3–6 resulted in a great number of articles. Two of the most recent articles were chosen as supporting articles. In the case of doubt of the relevance of a given article, the article was read thoroughly before deciding its relevance.

PubMed was searched in September 2016 for articles involving IL-6 and GI cancer published in English-language journals. The search was repeated in April 2018 to detect recently published studies. The primary purpose of the search was to identify clinical studies assessing the use of circulating IL-6 as a biomarker or as a therapeutic target in patients with GI cancer. The search combined these three areas shown in Figure 1, resulting in a total of 963 studies. Both medical subject heading and free text searching were performed. The articles were systematically assessed based on a subjective evaluation of the articles’ relevance and categorized into three groups: one group, including studies concerning IL-6 in patients with GI cancer, a second group, containing relevant background articles involving both reviews, *in vitro* and *in vivo* studies of IL-6 and other types of cancer, and a third group, containing excluded studies. Articles were excluded if IL-6 or cytokine were not mentioned in title or abstract and if the study was not based on patients with GI cancer, assessing IL-6 as a biomarker or a therapeutic target in GI cancer. This was done in two rounds. The first read-through was primarily based on title. Articles mentioning IL-6 and cancer in a diagnostic, prognostic or therapeutic context were included either in group one as primary articles with results entering this review’s results or in group two as supporting articles providing background information. The second read-through was based on an abstract or full text reading. In case of any doubt of the relevance of an article, the article was read thoroughly before deciding its relevance for the review. Only 48 articles were included in the results of this review, as only English, clinical studies concerning the clinical use of IL-6 in gastric cancer, bile duct cancer, pancreatic cancer and colorectal cancer were included. No murine studies were included in the results, however many of these studies are included as supporting articles. In addition, the reference lists of the included studies were searched for relevant studies.

Another search was done, including only randomized controlled trials. This search included all cancer types and resulted in 48 studies. Again, the relevance of the article was decided based on title and abstract. Furthermore, a search was done identifying supporting articles providing background information for each of the four cancer types. Using the Mesh-word option in PubMed, the search was limited to articles concerning humans while covering subjects such as anatomy, histology, classification, diagnosis, genetics, pathology, prevention and control and/or statistics and numerical data. This search included only articles that had the relevant cancer types mentioned in the title, and the search was narrowed down to include only articles published during the past 5 years, resulting in 8273 articles regarding gastric cancer, 236 regarding bile duct cancer, 7183 regarding pancreatic cancer and 2261 regarding colorectal cancer. Two of the most recent reviews concerning general knowledge of the cancer types were included as supporting articles in the review.

## RESULTS

### Diagnostic use of circulating IL-6 and correlation with clinical characteristics

34 articles investigated the diagnostic use of circulating (i.e. serum or plasma) IL-6 and the correlation between IL-6 and clinical characteristics. A tumor biomarker should be able to accurately detect malignancies or predict the behavior of the tumor or response to therapy [20, 22]. A diagnostic tumor biomarker is a biomarker used in differential diagnostics, which detects malignancy and separates the healthy subgroup from all patients with a given malignancy. This demands that a diagnostic biomarker must have a high sensitivity in order to detect all malignancies and a high specificity in order to avoid the false positive cases. In analyzing the results of the studies assessed in this section, a study was considered to investigate IL-6 as a diagnostic biomarker if a ROC-analysis was performed presenting either an AUC or sensitivity and specificity for increased IL-6 levels. A study was considered to evaluate the correlation between IL-6 and clinical characteristics in GI cancer if a test was performed for cancer specific findings such as lymph node metastases, invasion, distant metastases or tumor size. The test was considered positive if a *p*-value < 0.05 was presented.

### Gastric cancer

Six out of the 10 studies investigating gastric cancer and IL-6 (Table 1), assessed the correlation between clinical characteristics in patients with gastric cancer and IL-6, all showing a significant correlation. Ashizawa *et al.* [70] found significantly elevated IL-6 levels in gastric cancer patients compared to the healthy controls, and significantly elevated IL-6 was found in the presence of liver metastases, suggesting an association with tumor metastases. Ikeguchi *et al.* [67] found that high serum IL-6 correlated with the depth of tumor invasion, but not with lymph node metastases, and Kim *et al.* [79] found significant correlation between increased IL-6 and tumor size, tumor depth, lymph node metastases and stage. In a study by De Vita *et al.* [44] assessing both gastric and colorectal cancer patients as one cohort, IL-6 levels were shown to be significantly increased in patients with distant metastases and disseminated disease.

**Table 1:** Studies investigating IL-6 and gastric cancer

Gastric cancer									
Author	Year	No. of patients	Diag-nostic	C.C.	Prog-nostic	Cut-off (pg/ml)	AUC	Sens/Spec	Survival
**Sanchez-Zauco *et al.*** **[21]**	2017	162	+			3.2	0.599	97% 39%	
**Necula *et al.*** **[100]**	2012	51			+	38			*p = 0.022*^!^
**Szczepanik *et al.*** **[139]**	2011	99			+	290			NA^*^
**Lee *et al.*** **[140]**	2010	102		+		NA			
**Ikeguchi *et al.*** **[67]**	2009	90		+	+	0.8			5y survival: Low: 70% High: 48% *p = 0.49*
**Kim *et al.*** **[79]**	2009	115	+	+	−	6.8		86% 50%	3y survival: Low: 96% High: 81% *p = 0.01*^*!*^
**Liao *et al.*** **[69]**	2008	147			+	13			HR = 1.77^MV^ (1.07–2.92) *p = 0.026*
**Ashizawa *et al.*** **[70]**	2005	60		+	+	2			3y survival: Low: 87% High: 43% *p < 0.05*
**Kai *et al.*** **[66]**	2005	40		+	−	50			NA^!^
**Ilhan *et al.*** **[123]**	2003	42		+		NA			

^!^Kaplan Meier curve is presented in the paper.

^*^HR only presented for complications.

C.C. = clinical characteristics, AUC = area under the curve, Sens = sensibility, Spec = specificity.

Sanchez-Zauco *et al.* [21] assessed the diagnostic use of IL-6 in gastric cancer and found a high specificity (97%), but a low sensitivity (39%). In contrast to this, Kim *et al.* [79] found a high sensitivity (85.7%) and a low specificity (50.1%).

### Bile duct cancer

Only two studies focused on the correlation between clinical characteristics in patients with bile duct cancer and IL-6 (Table 2), and both included few patients. Both authors argue that high serum IL-6 levels may be useful in distinguishing between different neoplasms and between benignity and malignancy. Goydos *et al.* [72] performed a 3-year study assessing serum IL-6 in patients with bile duct cancer, and found that serum IL-6 was significantly higher in cholangiocarcinoma compared to patients with hepatocellular carcinoma, metastatic colorectal cancer and benign bile disease. A cut-off serum-IL-6 level above 100 pg/ml identified 80% of the bile duct cancer patients and excluded healthy adults, all patients with benign bile disease and 92% of the hepatocellular carcinoma patients. Cheon *et al.* [80] assessed the usefulness of serum IL-6 in bile duct cancer patients receiving photodynamic therapy (14 of the 26 patients), matching pre-therapy and post-therapy IL-6 levels to the tumor size. Serum IL-6 correlated with bile duct cancer tumor mass, which they used to define tumor burden, indicating that IL-6 has potential as a prognostic biomarker and could be useful in assessing the efficiency of treatment. However, Mott and Gores [81] commented that tumor mass is not identical to tumor burden in bile duct cancer.

**Table 2:** Studies investigating IL-6 and bile duct cancer

Bile duct cancer									
Author	Year	No. of patients	Diag-nostic	C.C.	Prog-nostic	Cut-off (pg/ml)	AUC	Sens/Spec	Survival
**Yoshitomi *et al.*** **[101]**	2012	25			+	NA			HR = 1.123 (1.008–1.252) *p = 0.035*
**Cheon *et al.*** **[80]**	2007	14	+	+		26 23		73%/92% 58%/100%	
**Goydos *et al.*** **[72]**	1998	15	+	+		100		Sens: 80%	

C.C. = clinical characteristics, AUC = area under the curve, Sens = sensibility, Spec = specificity.

### Pancreatic cancer

Three studies assessed the use of IL-6 as a diagnostic biomarker in patients with pancreatic cancer, all finding IL-6 useful (Table 3). Schultz *et al.* [26] studied a cohort of 559 patients and compared the use of serum IL-6 to CA 19.9 and found that IL-6 was no better than CA 19.9. More pancreatic cancer patients had elevated CA 19.9 than elevated IL-6. The area under the curve (AUC) for the diagnosis of pancreatic cancer was higher for CA 19.9 (AUC 0.94) compared to IL-6 (AUC 0.87). Both serum IL-6 and serum CA 19.9 were associated with tumor stage. Mroczko *et al.* [82] studied the diagnostic applicability of IL-6 in differentiating between pancreatic cancer and chronic pancreatitis and found that the AUC was higher for serum IL-6 (AUC 0.94) than serum CA 19.9 (AUC 0.86), CEA (AUC 0.89) and CRP (AUC 0.84).

**Table 3:** Studies investigating IL-6 and pancreatic cancer

Author	Year	No. of patients	Diag-nostic	C.C.	Prog-nostic	Cut-off (pg/ml)	AUC	Sens/Spec	Survival
**Kim *et al.*** **[74]**	2016	53		+	−	1.6			HR = 1.070 (0.830–1.378) *p = 0.602*
**Miura *et al.*** **[135]**	2015	79		+		1.2			
**Tsukinaga *et al.*** **[105]**	2015	7			−	2.0			p = 0.02^*^
**Arshad *et al.*** **[104]**	2013	32			+	NA			Low: 7.0 mths High: 3.1 mths *p = 0.009*
**Mitsunaga *et al.*** **[48]**	2013	60			+	1.9			HR = 2.10 (1.19–3.74)^MV^ *p = 0.11*
**Schultz *et al.*** **[26]**	2013	559	+		+	4.5	0.87		HR = 2.10 (1.11–3.60), *p = 0.011*^OP^ HR = 1.71 (1.33–2.20), *p < 0.0001*^NOP^
**Nixon *et al.*** **[103]**	2012	169			+	18			HR = 2.3 (1.7–3.2)
**Vizio *et al.*** **[106]**	2012	62		+	−	NA			HR = 1.002 (0.998–1.007) *p = 0.246*
**Mroczko *et al.*** **[82]**	2010	78	+	+	−	13	0.94 0.84^MA^		NA
**Bellone *et al.*** **[102]**	2006	41			+	NA			*p = 0.03*^!^
**Okada *et al.*** **[122]**	1997	55	+	+		3.0		55% 93%	

C.C. = clinical characteristics, AUC = area under the curve, Sens = sensibility, Spec = specificity.

MA = meta-analysis, OP = operated, NOP = non-operated.

^*^No correlation between IL-6 and OS > 1 year and OS < 1 year. Correlation between decrease in IL-6 during treatment and better survival.

### Colorectal cancer

Three studies investigated the use of serum IL-6 as a diagnostic biomarker in patients with colorectal cancer (Table 4). Xu *et al.* [83] performed a combined study in combination with a meta-analysis of prior studies that assessed the diagnostic and prognostic value of serum IL-6 in colorectal cancer. The data indicated that IL-6 might be a potential diagnostic biomarker that could be used to separate colorectal cancer patients from healthy subjects, reporting an AUC of 0.82 in a ROC analysis (95% CI 0.75–0.89). Using a cut-off level of 2.14 pg/ml, the sensitivity was 72% and specificity was 75%. In their meta-analysis, AUC was 0.79, sensitivity 72% (95% CI 46%-88%) and specificity 74% (95% CI 56%-86%). They concluded that due to the heterogeneity of the studies, their results should not be considered conclusive. In contrast to this study, Zhou *et al.* [14] preformed a meta-analysis of six published studies and found that there was no significant association between serum IL-6 levels and the relative risk of colorectal cancer. Groblewska *et al.* [24] demonstrated that IL-6 levels were significantly higher in colorectal cancer patients than in colorectal adenoma patients and healthy controls, and IL-6 levels proved to be a better diagnostic marker (AUC 0.90) than CEA (AUC 0.79), CRP (AUC 0.82) and CA 19.9 (AUC 0.65).

**Table 4:** Studies investigating IL-6 and colorectal cancer

Colorectal cancer									
Author	Year	Nr. of patients	Diag-nostic	C.C.	Prognostic	Cut-off (pg/ml)	AUC	Sens/Spec	Survival
**Chang *et al.*** **[65]**	2016	164		−	−	10			NA
**Hara *et al.*** **[109]**	2016	53			+	4.3			3y survival: Low: 71.4% High: 21.3% *p = 0.02*
**Thomsen *et al.*** **[107]**	2016	393			+	5.6			HR = 1.92 (1.56–2.37) *p < 0.001)*
**Xu *et al.*** **[83]**	2016	72	+		+	2.1	0.82 0.79^MA^	72% / 75%, 72% / 74%	HR = 1.76 (1.42–2.19)
**Olsen *et al.*** **[84]**	2015	189		−		NA			
**Hazama *et al.*** **[141]**	2014	17			+	2.0			HR = 4.21 (1.29–13.76) *p = 0.015*
**Reitter *et al.*** **[142]**	2014	726			+	NA			HR = 2.27 (1.227–4.371) *p = 0.009*
**Lee *et al.*** **[87]**	2013	77		+	−	9.1			OR = 0.26 (0.48–13.70) *p = 0.265*
**Liu *et al.*** **[108]**	2013	38			−	3.4			HR = 3.1 (1.4–6.6) *p = 0.004*^*A*^
**Shimazaki *et al.*** **[85]**	2013	46		+	+	2.4			HR = 4.1 (1.20–13.98) *p < 0.024*
**Kantola *et al.*** **[86]**	2012	148	+	+		NA	0.72		
**Eldesoky *et al.*** **[88]**	2011	35		+		6.7			
**Svobodova *et al.*** **[143]**	2011	174		−		NA			
**Kwon *et al.*** **[89]**	2010	132		−	−	12			HR = 1.391 (0.36–5.44) *p = 0.634*
**Yeh *et al.*** **[90]**	2010	99		−	+	10			HR = 0.403 (0.184–0.881) p = 0.023^*^
**Groblewska *et al.*** **[24]**	2008	76	+	+	−	13	0.90		OR = 1.005 *p = 0.402*
**Chung *et al.*** **[91]**	2006	106		+	(+)^T^	12			
**Dymicks-Piekarska *et al.*** **[92]**	2006	41		−		NA			
**Esfandi *et al.*** **[93]**	2006	50		+		NA			
**Nikiteas *et al.*** **[94]**	2005	74		−	+	8			*p < 0.05*^*!*^
**Chung *et al.*** **[95]**	2004	162		+	−	12			HR = 1.053 *p = 0.861*
**Belluco *et al.*** **[96]**	2000	208		+	+	10			RR = 1.820 (1.095–3.024) *p = 0.020* *5y survival* *Low: 69%* *High: 45%*
**Kinoshita *et al.*** **[97]**	1999	55		+		8.3			
**Ueda *et al.*** **[98]**	1994	24		+		3.1			

C.C. = clinical characteristics, AUC = area under the curve, Sens = sensibility, Spec = specificity.

MA = meta-analysis, T = only tested for tumor IL-6, A = HR represents a four-marker signature with low IL-6 as part of the high-risk group.

^!^Kaplan-Meier curve presented in the article.

^*^Tested for improved survival. HR also presented for 5 and 10 year survival, both significant.

Eighteen studies of patients with colorectal cancer [24, 65, 84–99] assessed the correlation between circulating IL-6 and clinical characteristics, reporting ambiguous results regarding tumor stage, resectability of the tumor, lymph node metastases, distant metastases, TNM stage and vascular invasion with 11 out of 18 articles finding correlation. In many of these studies, only some parameters proved significant, and due to the contradicting results, IL-6 and its correlation with clinical features remain elusive. De Vita *et al.* [44] assessed both gastric and colorectal cancer patients as one cohort, finding IL-6 levels to be significantly elevated in patients with distant metastases and disseminated disease.

### Prognostic use of circulating IL-6

Twenty-seven studies evaluated the prognostic use of circulating IL-6. A prognostic tumor biomarker is a test that indicates a high or a low risk of a cancer-related event, assuming that the patient receives no further treatment, if any [22]. A study was considered to investigate the prognostic use of IL-6, if the OS was investigated presenting either a Kaplan–Meier curve, hazard ratio (HR) or median survival times comparing patient groups with high and low serum IL-6 levels. To be considered positive, the test should result in a *p*-value < 0.05, a HR and confidential interval >1.00.

### Gastric cancer

Although the number of studies of patients with gastric cancer was small, all studies found a significant association between high serum IL-6 and short OS, suggesting a prognostic value in gastric cancer (Table 1). The most recent study performed by Necula *et al.* [100] showed an association between increased plasma IL-6 and tumor progression and between high tissue and plasma IL-6 levels and OS. Using a cut-off value of 1.97 pg/ml, Ashizawa *et al.* [70] found that OS was significantly longer in the low IL-6 level group than in the high IL-6 level group for both advanced gastric cancer and patients with lymphatic invasion. After 1 year, 69% of the gastric cancer patients with high IL-6 levels were alive compared to 94% of the gastric cancer patients with low serum IL-6, and after 3 years, the survival rates were 43% and 87%, respectively. Using multivariate analysis, Liao *et al.* [69] found a significant relation between high IL-6 and shorter OS (HR 1.77, 95% CI 1.07‒2.92). The gastric cancer patients with stage II/III and high IL-6 levels had a median survival of 618 days in contrast to 1418 days in patients with stage II/III and low IL-6 levels. Kai *et al.* [66] investigated IL-6 as a prognostic biomarker in patients with gastric cancer measuring IL-1β and IL-6, and found no significant difference in survival between patients with high tissue levels of IL-6 (cut-off level 50 pg/mg-protein) or IL-1β and low serum levels. Kim *et al.* [79] studied serum IL-6 and CRP in 115 gastric cancer patients undergoing gastrectomy, and found that high IL-6 was associated to short PFS and OS. In multivariate analysis, IL-6 was not related to TTP and OS. As expected, serum CRP correlated to IL-6, but CRP was not correlated to TTP and OS in univariate analyses.

### Bile duct cancer

Only one study has investigated the prognostic use of IL-6 in patients with biliary duct cancer (Table 2). In a phase II clinical trial, Yoshitomi *et al.* [101] found that increased IL-6 was associated with shorter OS (univariate analysis: HR 1.16, 95% CI 1.06‒1.27; multivariate analysis: HR 1.12, 95% CI 1.01‒1.25).

### Pancreatic cancer

Nine studies have assessed the use of IL-6 as a prognostic biomarker in patients with pancreatic cancer (Table 3). Five studies showed IL-6 to be useful [26, 48, 102, 104]. Schultz *et al.* [26] found that high IL-6 in pancreatic cancer patients was associated with short OS, and in multivariate analyses, the HR was 1.71 (95% CI 1.33–2.20) for high serum IL-6 and 1.54 (95% CI 1.06–2.24) for high serum CA 19.9 in patients with locally advanced or metastatic pancreatic cancer. In patients who had undergone surgery, the HR was 2.03 (95% CI 1.11–3.70) for high serum IL-6 and 2.51 (95% CI 1.22–5.15) for high CA 19.9 levels. The combination of high CA 19.9 and high IL-6 identified pancreatic cancer patients with a very short median survival of only 7.5 months compared to 34.4 months for pancreatic cancer patients with normal levels of CA 19.9 and IL-6. Thus, IL-6 appears to be a better prognostic biomarker than CA 19.9 in non-operable pancreatic cancer patients, but CA 19.9 is a better prognostic biomarker for pancreatic cancer patients who undergo surgery. Mitsunaga *et al.* [48] assessed the prognostic value of IL-6 and IL-1β in patients with pancreatic cancer receiving gemcitabine, and using multivariate analysis they found that high IL-6/high IL-1β levels were an independent prognostic factor for poor OS (HR = 2.10) and short PFS (HR = 2.32). In patients only presenting high IL-6 levels, there was an association with short PFS (*p* = 0.013), but not for OS (*p* = 0.053). In a study performed using serum from patients participating in a phase II clinical trial assessing the tumor response in patients with advanced pancreatic cancer receiving gemcitabine and IV omega-3 rich lipid emulsion, Arshad *et al.* [104] showed that low IL-6-levels correlated with improved OS, with a median OS of 7 months vs. 3.5 months for patients with high IL-6 levels. Changes in IL-6 levels were, however, not useful for monitoring treatment.

Kim *et al.* [74] assessed the serum IL-6 in patients with pancreatic cancer, including only patients with no or fewer liver metastases. At the last follow-up, the patients were separated into a limited and a progressed group (liver metastases), with IL-6 levels proving to be significantly higher in the progressed group (2.0 versus 1.4). However, they found no correlation between high serum IL-6 and OS (HR = 1.07, 95% CI 0.83–1.38). In a small study by Tsukinaga *et al.* [105], plasma IL-6 prior to treatment with chemoimmunotherapy was not associated to OS, however the decrease in IL-6 during treatment was associated with better survival (*p* = 0.02). Mrozcko *et al.* [82] and Vizio *et al.* [106] did not find that serum IL-6 levels in patients with pancreatic cancer correlated with shorter OS using multivariate analyses.

### Colorectal cancer

Sixteen studies assessed the use of serum IL-6 as a prognostic biomarker in patients with colorectal cancer with contradicting results (Table 4). Nine of the studies found that serum IL-6 was an independent prognostic biomarker of OS. Thomsen *et al.* [107] measured serum IL-6 and CRP in 393 patients with metastatic colorectal cancer receiving first line chemotherapy. For patients with high serum IL-6, the median PFS was 7.7 months and the OS 16.6 months compared to 8.9 months (PFS) and 26 months (OS) for patients with low serum IL-6. This significance remained after adjustment for other prognostic biomarkers and clinical characteristics. Xu *et al.* [83] performed a meta-analysis of 10 studies investigating the prognostic use of IL-6. They found that the pooled HR was 1.76 (95% CI 1.42–2.19), indicating that high serum IL-6 in colorectal cancer patients is a predictor of short OS. No significant heterogeneity was reported. In this context, Yeh *et al.* [90] showed promising results, as serum IL-6 proved to be an independent prognostic factor. Using a cut-off level of 10 pg/ml, the OS of patients with serum IL-6 >10 pg/ml was significantly shorter after 3 years (HR 0.40, 95% CI 0.18–0.88), 5 years (HR 0.37, 95% CI 0.20–0.70) and 10 years (HR 0.42, 95% CI 0.25–0.73).

The following studies demonstrated no correlation between increased serum IL-6 and OS in patients with colorectal cancer. Many of these studies were large, with a total of 649 patients as compared to 1794 patients in the positive studies. Chang *et al.* [65] measured serum IL-6, IL-1β, TNF-α and CRP, and used median levels of each cytokine as cut-off level (10 pg/ml, 10 pg/ml, 55 pg/ml and 5 mg/l, respectively). Assessing only PFS, they found a tendency to longer PFS in patients with increased serum IL-6, but multivariate analysis showed that only tumor stage was an independent marker of PFS. High serum levels of CRP were associated to a high cytokine intensity of IL-1β, TNF-α and IL-6, but no correlation between CRP and IL-6 was found. Liu *et al.* [108] did not find IL-6 to be an independent prognostic factor; however, they did find that it correlated with outcome in combination with three other biomarkers (Ang-2, IGFBP-3 and VCAM-1).

### Meta-analysis of the prognostic use of IL-6 in pancreatic and colorectal cancer

**Figure 2:**
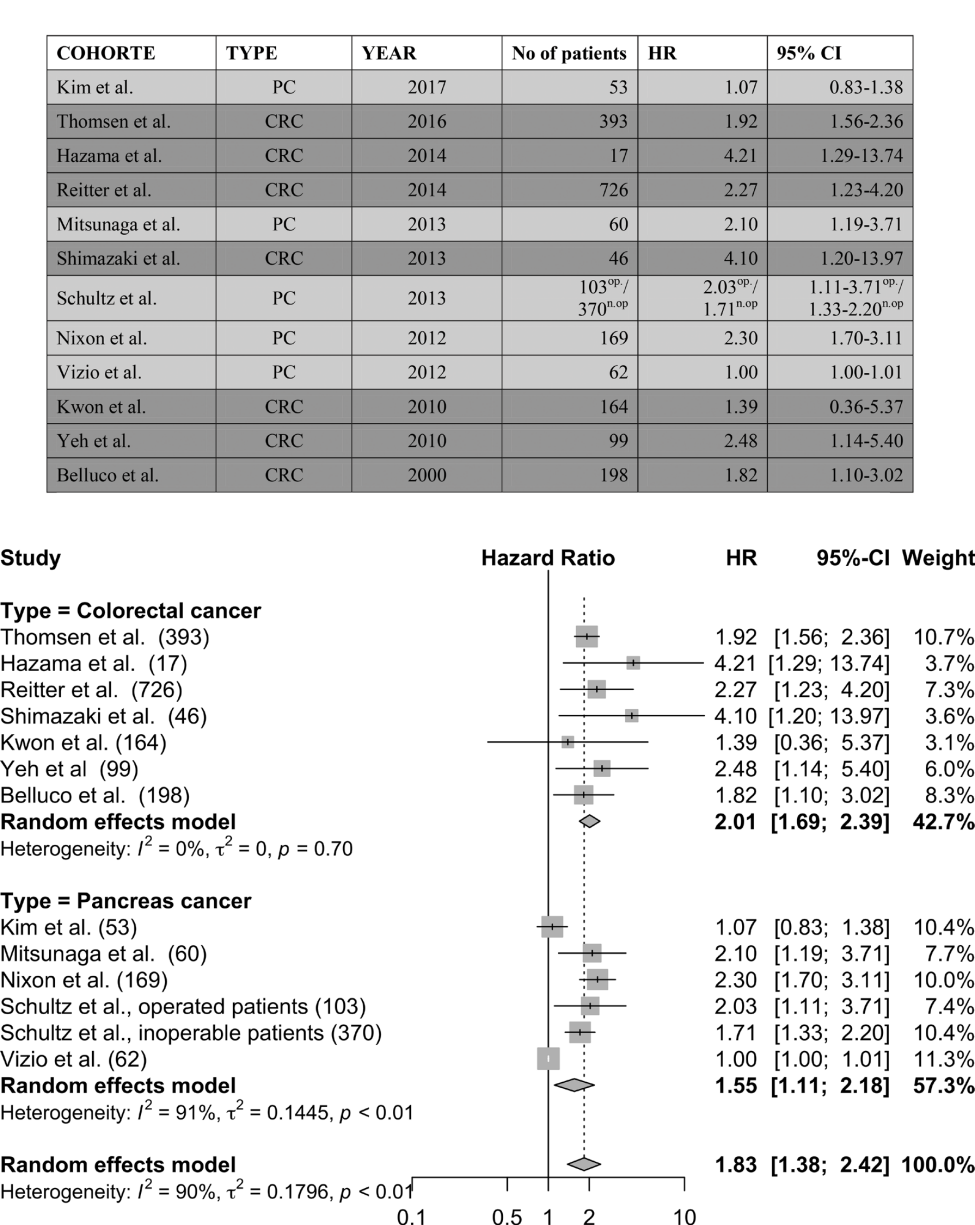
The meta-analysis was performed on selected studies concerning colorectal cancer and pancreatic cancer op = operated patient cohort, n.op = non-operated patient cohort.

In order to clarify the prognostic value of IL-6 in pancreatic cancer and colorectal cancer, we undertook a meta-analysis of the two cancer types, see Figure 2. Several studies assessed the relationship between IL-6, pancreatic cancer and colorectal cancer. However, the studies included in this meta-analysis are limited to studies presenting data with HRs and confidence intervals, thereby narrowing the number of articles included. It should be noticed that different cut-off levels for high and low levels of circulating IL-6 were used in the studies included in meta-analysis. Furthermore, different follow-up periods and ELISA measurement to determine circulating IL-6 levels were used, resulting in a significant heterogeneity between the studies in terms of both different methodologies and clinical approaches.

In conclusion, five studies were included in the meta-analysis concerning pancreatic cancer, representing 817 patients. The pooled HR for pancreatic cancer was 1.55 (95% CI 1.11‒2.18). Seven studies were included in the meta-analysis concerning colorectal cancer, representing 1,643 patients. The pooled HR for colorectal cancer was 2.01 (95% CI 1.69‒2.39).

### Predictive use of circulating IL-6

A predictive biomarker is a biomarker which estimates the relative likelihood that a cancer will respond to a specific treatment prior to treatment [22]. In investigating a biomarker, a study should include a homogenous group of patients randomized to receive either standard treatment or the specific treatment assessed in the study. The pre-treatment biomarker should foresee which patients respond to the treatment by comparing OS, PFS or response rate. In this review, a study was considered to investigate the predictive use of circulating IL-6, if the study assessed how the circulating IL-6 levels could identify the group benefitting from treatment in two homogenous groups of GI cancer patients receiving different treatments. No studies were performed in patients with gastric cancer, biliary duct cancer or pancreatic cancer. Only one study by Hara *et al.* [109] assessed the predictive use of serum IL-6 in colorectal cancer (Table 4), showing IL-6 to be useful as a predictive biomarker.

They assessed the effect of IL-6 on outcome and the response to therapy in patients with advanced or metastatic colorectal cancer treated with chemotherapy or chemotherapy and bevacizumab combined. High IL-6 was associated with short PFS in multivariate analysis (RR 2.1, 95% CI 1.3–3.6). Using the cut-off value 4.3 pg/ml, patients with high IL-6 had significantly shorter 3-year OS than those with low IL-6 levels (21% vs. 71%). The patients with highest IL-6 levels also had significantly shorter median PFS (HR = 2.6, 95% CI 1.5–4.8).

## DISCUSSION

Hayes [22] highlights that a tumor biomarker test should be used in clinical care only when there is analytic validity, clinical validity and clinical utility, meaning that the measurement should be accurate and reliable and identify a defined disorder or separate a population into groups with distinct clinical outcomes. In addition, the use of the test results should improve measurable clinical outcomes [110]. It has, however, been difficult to achieve a satisfying level of evidence due to a lack of adequate funding, inappropriately designed studies, non-publication bias and lack of guidelines when approving new biomarkers [111]. Many initiatives have been taken to overcome these difficulties. The REMARK (reporting recommendation for tumor biomarker prognostic studies) guidelines attempt to increase the transparency in information provided by studies investigating possible tumor biomarkers [111]. The REMARK criteria consist of 20 items to report, including research goals, hypotheses, patient characteristics, assay and measurement methods, statistical design and study results [112]. Furthermore, it has been suggested that due to the low level of evidence most tumor biomarker studies achieve, multiple studies must be done to validate the usefulness of a tumor biomarker [111].

In this review, we assessed the diagnostic and prognostic value of circulating IL-6 in patients with GI cancer. Many patients with GI cancer have elevated IL-6 levels, but only a few studies have taken into consideration that IL-6 is increased in serum or plasma in patients with other types of cancer and other diseases characterized by inflammation. IL-6 is part of the systemic inflammatory condition following cancer and since IL-6 is produced both by cancer cells and inflammatory cells, circulating IL-6 may be a biomarker for both inflammation and tissue remodeling, and an increase in serum IL-6 is also found during acute or chronic stress (e.g. surgical procedures, sepsis, inflammatory bowel disease, trauma), and many other cancer types, e.g. hepatocecullar carcinoma [25, 72], lung [113] breast [114], cervical [115], oesophageal [116], head and neck [117, 118], ovarian [119], prostate [120], renal cancer [121] and hematological diseases [72]. Hence, IL-6 is not cancer specific for a single type of cancer.

Regarding the results of this review, some studies find equivocal results when investigating tumor depth invasion or lymph node metastases, but the overall picture seems to indicate that high levels of IL-6 are associated with severe clinical features of patients with GI cancer. The majority of the studies found a difference in serum IL-6 between patients with GI cancer and healthy subjects, although only few studies investigated the diagnostic use of IL-6 properly. Given that IL-6 is not cancer specific, the use of IL-6 as a diagnostic biomarker is challenging. A biomarker test is used to identify abnormalities in patients, and the biomarker should reflect the presence of the abnormality [22]. The use of IL-6 as a diagnostic tumor biomarker demands close attention to the cut-off level. A good diagnostic tumor biomarker detects all patients with a given malignancy (high sensitivity) and rules out all healthy subjects (high specificity). Hence, the cut-off level of IL-6 as a tumor biomarker must be sufficiently high in order to only diagnose the patients with malignancies and not patient with inflammatory conditions, and still relatively low in order to detect all patients with malignancies. In the studies assessed in this review, sensitivity and specificity were not convincingly high, nor superior to other diagnostic methods such as serum CEA and CA 19-9, and many cancers would not be detected with IL-6 as the diagnostic biomarker. Although the use of IL-6 as an independent diagnostic tumor biomarker seems doubtful, it may be useful as a quick biomarker test in general practice. Patients with GI cancers are often diagnosed at advanced stages due to unspecific symptoms. High serum IL-6 levels in a subject with weight loss and fatigue, but without any signs of infections or inflammatory disease, would suggest a need for a CT-scan, since the risk of cancer is high if serum IL-6 is very high. This has to be studied in future studies of patients referred to hospital in suspicion of cancer.

The results of the studies investigating IL-6 as a prognostic biomarker in patients with GI cancer, require a differentiation between the four GI cancers. The studies of patients with gastric cancer and biliary duct cancer were few and included too few patients to be conclusive. Several of the studies with pancreatic cancer and colorectal cancer tested a large number of patients and found a significant association between high circulating IL-6 levels and short OS. The studies concerning colorectal cancer were however ambiguous. Since the colorectal cancer studies involved a large number of patients showing different results, the results regarding colorectal cancer and IL-6 seems inconclusive. The results of the meta-analysis do, however, show IL-6 to be significant as a prognostic biomarker for OS in colorectal cancer. For pancreatic cancer, the results of the studies assessed were unanimous showing a significant association between high circulating IL-6 and short OS, which was confirmed in the meta-analysis. This suggests that IL-6 could be a useful prognostic biomarker for patients with pancreatic cancer. However, several of these studies showed that IL-6 had a prognostic value similar to that of the routine biomarkers serum CA 19-9 and CEA, so the use of IL-6 to provide independent information regarding prognosis is still unknown.

It would be interesting to investigate the potential role of IL-6 as a prognostic biomarker for both early (stage I and II) and late (stage III and IV) GI cancer. This has, however, not been possible with the current available studies, since only few studies differentiated between patients presenting early and late cancer stages. It is interesting that Schultz *et al.* [26] found that IL-6 appears to be a better prognostic biomarker than CA 19.9 in non-operable pancreatic cancer patients, but CA 19.9 is a better prognostic biomarker for pancreatic cancer patients who undergo surgery. For gastric cancer, Liao *et al.* [69] found IL-6 to be prognostic for stage III and IV, but not for stage I and II, and for colorectal cancer, Shimazaki *et al.* [85] found a significant difference in IL-6 levels between stage 0-II and stage III-IV. Also, we observed a common trend showing a significant correlation between median serum IL-6 and increasing cancer stages [21, 24, 26, 67, 74, 79, 82, 88, 91, 93, 95, 96, 98, 102, 106, 122, 123], which indicate that IL-6 may be a marker for a more severe type of cancer. Future studies should therefor differentiate between early and late cancer stages when investigating the use of IL-6 as a prognostic biomarker.

Performing a systematic review regarding circulating IL-6 in patients with GI cancer in which data from multiple studies are evaluated and compared provides us with a higher level of evidence. Comparing the data has, however, proved to be a challenge. More than 200.000 articles has been published regarding gastric-, pancreatic-, bile duct- and colorectal cancer, however it is interesting that only 48 articles assessed the clinical use of IL-6 as a biomarker. The limited amount of data on IL-6 and GI cancer combined with the fact that the studies did not publish all data and the discrepancy in what data is considered important to include in the articles, have resulted in a hampered comparison of the data. Furthermore, studies using different cut-off values of IL-6, focusing on multiple end-points, not reporting negative results and failure to follow the REMARK criteria proposed results that fell short of the desired transparency needed to make a systematic review with a high level of evidence. In the future, studies dealing with biomarkers should follow international reporting guidelines such as the REMARK criteria to increase the level of evidence and ease the comparison of results.

The role of the inflammatory response in cancer has become more appreciated during the past few years [50]. Today, tumor-promoting inflammation is seen as an enabling mechanism that plays a role in many of the hallmarks of cancer, thereby promoting the growth of cancer [51]. The inflammatory cells, particularly macrophages [42], enhance tumor growth by secreting important growth factors into the tissue surrounding the tumor, stimulating angiogenesis, invasion, proliferation and cell survival, putting the tumor cell in an anti-apoptotic, expansive and proliferative state, all of which affects therapeutic resistance and a poorer prognosis [50, 51, 124]. Since immune cell infiltration can be both tumor-promoting and tumor-suppressing, total elimination of the inflammatory response is not desirable, and modern medicine must learn to influence the tumor microenvironment in order to attract and differentiate the immune infiltrate that will produce a tumor-suppressing environment [125]. Myeloid cells show a great deal of plasticity, being very sensitive to signaling compounds surrounding them, and therefore, by blocking some of these tumor-promoting signals – for example IL-6, which alters this steady-state – the tumor-microenvironment can be directed into exerting an anti-tumor action [125]. IL-6 has been shown to inhibit the tumor suppressing immune cells in colorectal cancer cells by down-regulating the activity of cytotoxic T-lymphocytes through suppression of the maturation of dendrite cells [37]. In IL-6 deficient mice injected with colon cancer cells, tumor growth was decreased and dendrite cells, helper T cells and cytotoxic T-cells were increased in the tumor microenvironment [126]. IL-6 is part of the inflammatory response in many types of cancer, but since it is only a small piece of a very complex interaction between multiple inflammation mediators and a large number of different cells, foreseeing the outcome of treating cancer patients with anti-IL-6 therapeutics is difficult [30, 36].

Investigations of the use of IL-6 as a therapeutic target in patients with GI cancer have not yet been performed. However, IL-6 has been shown to have a proliferative effect on gastric cancer cells [68], and it is also an important growth factor for neoplastic biliary duct cancer [60, 62]. Inhibition of the activation of STAT3, e.g. by the anti-IL6R monoclonal antibody Tocilizumab, is known to down-regulate the immune suppression caused by tumor cells [41], and inhibition of IL-6 signaling may be a therapeutic for patients with cancer when IL-6 is over-expressed [28]. Tocilizumab has been used since 2010 for treatment of patients with severe rheumatoid arthritis [45]. However, in small clinical trials regarding cancer, treatment with anti-IL6 or anti-IL6R has shown no clinical effect in patients multiple myeloma [127, 128], metastatic renal cell cancer [129] and castration-resistant prostate cancer [47]. In patients with recurrent epithelial ovarian cancer, a phase I clinical trial recently showed an acceptable safety profile and a possible immunological benefit when they were treated with Tocilizumab in combination with chemo-therapy [41]. A phase II study is now ongoing, testing Tocilizumab in combination with chemo-therapy in patients with endothelial ovarian cancer [130], and a phase II study is ongoing evaluating whether gemcitabine/nab-paclitaxel combined with Tocilizumab is more effective than gemcitabine/nab-paclitaxel alone in patients with locally advanced or metastatic pancreatic cancer with elevated serum CRP [130]. So far, treatment with Tocilizumab has proved to be well tolerated, with a low frequency of side-effects [41, 47, 129]. Mitsunaga *et al.* [131] studied nine patients with advanced pancreatic cancer who received gemcitabine in combination with Tocilizumab and found no clinical benefit, but it was a small study and no group treated with only gemcitabine was included. The cell studies described for the four types of GI cancer (gastric cancer, biliary duct cancer, pancreatic cancer and colorectal cancer) provide a solid base for documenting the impact of IL-6 in the development, growth and invasion of these cancers. The use of IL-6 as a therapeutic target is currently being investigated for pancreatic cancer, but trials should be done for the three other cancers types, especially since inhibition of anti-IL-6R with Tocilizumab seems to be well tolerated. Another anti-IL-6 drug, Siltuximab, binds to circulating IL-6 preventing it from binding to its receptor, and has also proven to be well tolerated [28, 30, 132]. Although promising in theory, studies have shown that Siltuximab and IL-6 forms circulating complexes, which are unable to be cleared by the digestion of cells and renal clearance, resulting in a drastic increase in serum IL-6 level when ending the treatment [30]. Another way to inhibit the effect of IL-6 could be to decrease the level of circulating IL-6. In a meta-analysis by Yu *et al.* [133], they studied the effect of omega-3 polyunsaturated fatty acids on patients undergoing surgery for GI cancers. The meta-analysis showed that serum IL-6 levels decreases following omega-3 fatty acids supplementation versus isocaloric nutrition. The supplement seemed to decrease the level of inflammation and increase immune function by modulating the cells of the immune system. This indicates that an attempt to target IL-6 when treating patients with GI cancer should incorporate different approaches, including both treatment with an anti-IL-6R to inhibit the effect of IL-6 and an attempt to lower the circulating levels of IL-6, for example through nutritional means.

The idea of targeting IL-6 in cancer treatment is also relevant since a systemic inflammatory response is associated with a poorer outcome, independent of tumor stage [50]. IL-6 has been suggested as treatment in combination with immunotherapy such as immune checkpoint therapies, cancer peptide vaccines or immunological adjuvants, since IL-6 seems to downregulate the tumor-suppressing tumor micro-environment [37, 126] and has been tested with promising results in murine studies [75]. A strong systemic inflammatory response in cancer patients correlates with cancer-associated symptoms such as cachexia, anorexia, lowered physical function, fatigue, pain and depression. Although clear evidence is lacking, lowering the systemic immune response could thereby increase the day-to-day quality of life of many patients with cancer [30, 50].

CRP is currently used as a general biomarker of systemic inflammation and IL-6 induces the secretion of CRP from the hepatocytes [28, 92, 134]. Several of the studies investigated in this review assessed the correlation between serum IL-6 and CRP levels in cancer patients, and the majority found a significant correlation between IL-6 and CRP. This has also been reported for patients with lung cancer [113], colorectal cancer [86, 92, 94, 95, 107], gastric cancer [67, 79], pancreatic cancer [48, 74, 122, 135]. CRP and IL-6 also correlated to cytokine intensity in gastric cancer [65] and certain clinical characteristics such as more advanced tumor stage in gastric [65, 79, 123] and colorectal cancer [92], shorter survival in colorectal [24, 94, 95], PC [135] and lung cancer [47], and cachexia in patients with colorectal [94], pancreatic [24, 82] and lung cancer [113]. CRP has been associated to paraneoplastic symptoms and cachexia, leading to an understanding of a condition with chronic elevated IL-6 in cancer which leads to anemia, elevated CRP levels and a change in the gut microbiota that possibly increases the gut permeability further worsening the cachexia [136]. According to a large study by Thomsen *et al.* [137], a high serum level CRP and IL-6 was associated to a severely impaired health-related quality of life compared to patients with normal levels in 512 patients with metastatic colorectal cancer patients receiving first line chemotherapy with or without Cetuximab.

In patients with pancreatic cancer, treatment with anti-IL-6R could potentially result in an amelioration of the severe symptoms from which many patients with metastatic pancreatic cancer suffer [122]. High serum IL-6 levels in pancreatic cancer patients are associated with symptoms such as anemia, metastasis, fatigue and most important cachexia, which is a severe complication for many of these patients [12, 26, 122, 138]. This is why a number of studies, e.g. Miura *et al.* [135] suggest the use of treatment with an anti-IL-6R antibody in treatment of patients with metastatic pancreatic cancer. They found that high IL-6 was associated with impairments in general activity and working ability. It has also been discussed whether or not the chemo-resistance of the GI cancer cells is dependent on IL-6 [48]. Since the literature reveals a poorer response to chemo-therapy when IL-6 is increased, evaluation of IL-6-levels might help in the development of personalized treatment strategies for patients with pancreatic cancer [26, 82]. Hence, even if targeting IL-6 in patients with GI cancer proves not to have any anti-tumor effect, the treatment may decrease severe cancer symptoms such as cachexia and fatigue, improving the patients’ quality-of-life [136].

## CONCLUSIONS

This systematic review found 48 publications investigating IL-6 as a cancer biomarker in 5316 patients with gastric, bile duct, gallbladder, pancreatic and colorectal cancer. The overall picture of IL-6 was that this cytokine was elevated in plasma or serum from patients with these types of GI cancer compared to healthy controls. We assessed the diagnostic value of IL-6 in the hope of finding methods to detect the GI cancers earlier, but IL-6 has little value as independent single diagnostic biomarker, since it is not cancer specific and not sensitive enough. However, IL-6 might be useful as a bed-side test in general practice, possibly identifying patients with unspecific or vague symptoms of cancer. In the majority of the studies, serum IL-6 correlated with the clinical characteristics of the patients.

The use of IL-6 as a prognostic biomarker seems promising for patients with gastric-, pancreatic-, and mayble also for bile duct cancer. The results regarding the prognostic use of IL-6 in colorectal cancer were unfortunately too equivocal to allow firm conclusions. In some of the studies, IL-6 did not provide additional information compared to serum CEA and CA 19-9.

Cancer cell studies have shown a correlation between IL-6 and chemo resistance and increased proliferation, and the use of IL-6 as a therapeutic target is currently being investigated. Targeting IL-6 may improve OS in patients with GI cancer and decrease severe cancer symptoms like fatigue and cachexia and improving quality of life in these patients.

## Abbreviations

GI: gastrointestinal cancer
IL: interleukin
OS: overall survival
CEA: carcinoembryonic antigen
CA 19-9: carbohydrate antigen 19-9
miRNA: micro-RNA
mIL-6R: Membranous IL-6 receptor
sIL-6R: Soluble IL-6 receptor
R: receptor
CI: confidence interval
HR: hazard ratio
ELISA: enzyme-linked immunosorbent assay
p: *p*-value
GC: gastric cancer
CRC: colorectal cancer
PC: pancreatic cancer
CCA: cholangiocarcinoma
